# Mechanistic Study on the Corrosion of (La,Sr)(Co,Fe)O_3-δ_ Cathodes Induced by CO_2_

**DOI:** 10.3390/molecules28227490

**Published:** 2023-11-08

**Authors:** Na Xu, Shijiao Zhang, Qiongyu Zhou, Hairui Wang, Lina Zhao, Zhanlin Xu

**Affiliations:** 1 Key Laboratory of Preparation and Application of Environmental Friendly Materials, Ministry of Education, Jilin Normal University, Changchun 130103, China; xuna8912@126.com (N.X.); zhangshijiao2023@126.com (S.Z.);; 2 Department of Chemistry, Jilin Normal University, Siping 136000, China; 3 School of Materials Science and Hydrogen Energy, Foshan University, Foshan 528000, China

**Keywords:** CO_2_ corrosion, (La,Sr)(Co,Fe)O_3-δ_ cathodes, stability study, degradation mechanisms

## Abstract

Solid Oxide Fuel Cell (SOFC) cathodes operating in ambient atmospheric conditions inevitably encounter CO_2_ contamination, leading to sustained performance deterioration. In this investigation, we examined the impact of CO_2_ on three variants of (La,Sr)(Co,Fe)O_3-δ_ cathodes and employed the distribution of relaxation times method to distinguish distinct electrochemical processes based on impedance spectra analysis. We meticulously analyzed and discussed the corrosion resistance of these (La,Sr)(Co,Fe)O_3-δ_ cathodes under high CO_2_ concentrations, relying on the experimental data. Electrochemical impedance spectroscopy results revealed that La_0.6_Sr_0.4_Co_0.2_Fe_0.8_O_3-δ_ (LSCF−6428), La_0.4_Sr_0.6_Co_0.2_Fe_0.8_O_3-δ_ (LSCF−4628), and La_0.4_Sr_0.6_Co_0.2_Fe_0.7_Nb_0.1_O_3-δ_ (LSCFN−46271) cathodes exhibited persistent degradation when exposed to CO_2_ at temperatures of 650 °C or 800 °C during the durability-testing period. An increase in electrode polarization resistance was observed upon CO_2_ introduction to the electrode, but electrode performance recovered upon returning to a pure air environment. Furthermore, X-ray diffraction and scanning electron microscopy analyses confirmed that CO_2_ did not cause permanent damage to the (La,Sr)(Co,Fe)O_3-δ_ cathodes. These findings indicate that the (La,Sr)(Co,Fe)O_3-δ_ cathodes exhibit excellent resistance to CO_2_-induced corrosion.

## 1. Introduction

Solid oxide fuel cells (SOFCs) stand as the most efficient devices devised to date for the direct conversion of chemical fuels into electrical power [1,2,3]. Nevertheless, despite several decades of development, long-term stability remains a formidable obstacle impeding the widespread commercialization of SOFC technology [4]. Among the crucial components of the SOFC, the cathode assumes a pivotal role, with its overpotential often accounting for a substantial portion of energy loss. Ensuring the chemical and morphological stability of electrode materials under the demanding operational conditions of SOFCs remains of paramount significance [5,6,7].

In both research and practical applications, ambient air is typically directed straight to the cathodes. This air carries contaminants, such as CO_2_, which are challenging to eliminate and contribute to cathode corrosion and degradation [8]. Consequently, an in-depth investigation into the effects of CO_2_ on the performance and durability of SOFC cathodes becomes imperative.

CO_2_ readily engages in reactions with alkaline-earth elements present in cathode materials, leading to the formation of carbonates [9,10]. Consequently, the Ba-based Ba_0.5_Sr_0.5_Co_0.8_Fe_0.2_O_3-δ_ (BSCF) cathode, recognized for its superior performance, demonstrates limited resilience to CO_2_ due to the presence of Ba and Sr as A-site alkaline-earth elements [11,12]. Yi, Wang, and Yang reported that the introduction of partial Co substitution with Fe or Nb, yielding cathodes such as BaCo_0.4_Fe_0.4_Nb_0.2_O_3-δ_, Ba_0.5_Sr_0.5_Co_0.8_Fe_0.1_Nb_0.1_O_3-δ_, and Ba_0.9_Co_0.7_Fe_0.2_Nb_0.1_O_3-δ_, substantially enhances corrosion resistance against CO_2_ [13,14,15]. Investigations into the effects of CO_2_ on La-based cathodes, such as La_0.8_Sr_0.2_MnO_3-δ_, La_0.6_Sr_0.4_CoO_3-δ_, and La_0.6_Sr_0.4_FeO_3-δ_, have also been systematically conducted [16,17]. Notably, the absence of Ba in La-based cathodes results in improved tolerance to CO_2_. It is worth noting that the CO_2_ corrosion mechanism varies among different cathodes due to the diversity in cathode chemical composition and crystal structure [18].

(La,Sr)(Co,Fe)O_3-δ_ cathodes have garnered considerable attention due to their exceptional catalytic activity, mixed electronic–ionic conductivity, and compatibility with other cell components, both chemically and mechanically. Within the diverse spectrum of (La,Sr)(Co,Fe)O_3-δ_ cathode compositions, La_0.6_Sr_0.4_Co_0.2_Fe_0.8_O_3-δ_ (LSCF−6428) stands out as a favored choice among IT-SOFC developers [19,20,21]. The impact of atmospheric CO_2_ on surface segregation and phase formation in LSCF−6428 was investigated [22,23]. The findings revealed that the presence of atmospheric CO_2_ accelerated the kinetics of Sr surface segregation, primarily due to the heightened thermodynamic driving force for SrCO_3_ formation. Nevertheless, the crystal structure and performance of both La_0.6_Sr_0.4_FeO_3-δ_ and La_0.6_Sr_0.4_Fe_0.8_Nb_0.2_O_3-δ_ materials remained largely unaffected under a CO_2_ atmosphere, demonstrating their robust CO_2_ tolerance [17,24].

Previous research has demonstrated that to further enhance the performance of LSCF−6428, the partial substitution of La by Sr at the A-site and the addition of a small amount of Nb to the B-site can improve its electrical conductivity and chemical stability [25,26,27,28]. Consequently, the resultant La_0.4_Sr_0.6_Co_0.2_Fe_0.8_O_3-δ_ and La_0.4_Sr_0.6_Co_0.2_Fe_0.7_Nb_0.1_O_3-δ_ cathodes contain a higher proportion of Sr element. Therefore, it becomes imperative to investigate CO_2_ corrosion on these two cathode variants and compare them with the state-of-the-art LSCF−6428 cathode.

In this study, we delve into the mechanisms of CO_2_-induced corrosion affecting La_0.6_Sr_0.4_Co_0.2_Fe_0.8_O_3-δ_, La_0.4_Sr_0.6_Co_0.2_Fe_0.8_O_3-δ_, and La_0.4_Sr_0.6_Co_0.2_Fe_0.7_Nb_0.1_O_3-δ_ cathodes.

## 2. Results and Discussion

### 2.1. Impedance and DRT Analysis of Three Electrode Configurations under Various Oxygen Partial Pressures

**Figure 1:**
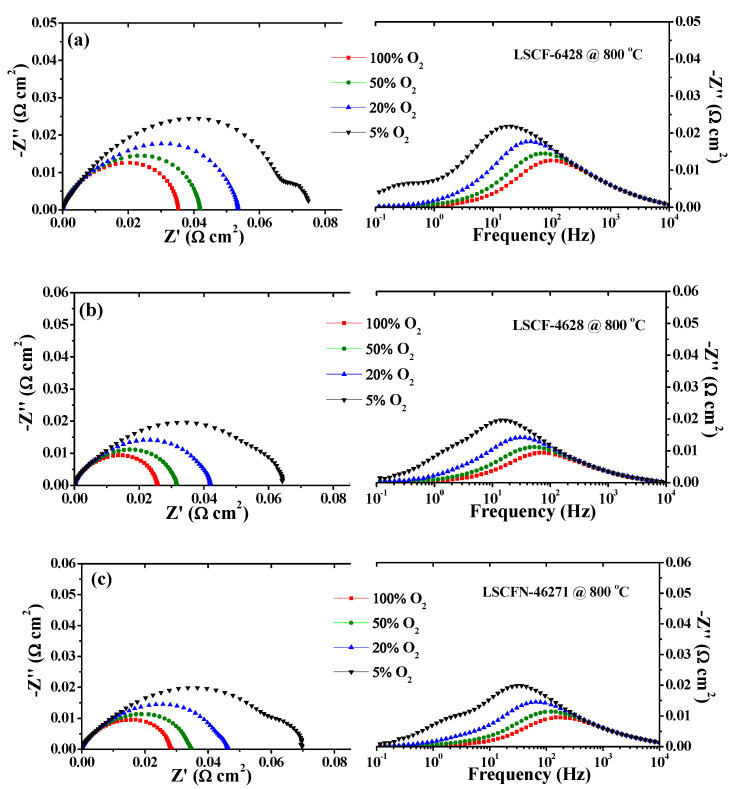
Nyquist plots and Bode plots for (**a**) LSCF−6428, (**b**) LSCF−4628, and (**c**) LSCFN−46271 electrodes measured at 800 °C under different oxygen partial pressures in O_2_ + N_2_ mixtures.

To determine the frequency range associated with various electrochemical processes of oxygen electrode reactions, impedance spectra Nyquist plots and Bode plots were obtained for LSCF−6428, LSCF−4628, and LSCFN−46271 symmetric electrodes at 800 °C (see additional data in Supplementary Materials). These electrodes were exposed to four different oxygen partial pressure atmospheres, as depicted in Figure 1: 5% O_2_-95% N_2_, 20% O_2_-80% N_2_, 50% O_2_-50% N_2_, and 100% O_2_. From the Nyquist plots, it can be observed that the polarization impedance of the symmetric electrodes increases with decreasing oxygen partial pressure. The same conclusion can be drawn from the modulus curves of impedance in the Bode plots. Additionally, the Bode plots illustrate changes in the phase angle within specific frequency domains, showing that significant variations occur primarily below 400 Hz.

**Figure 2:**
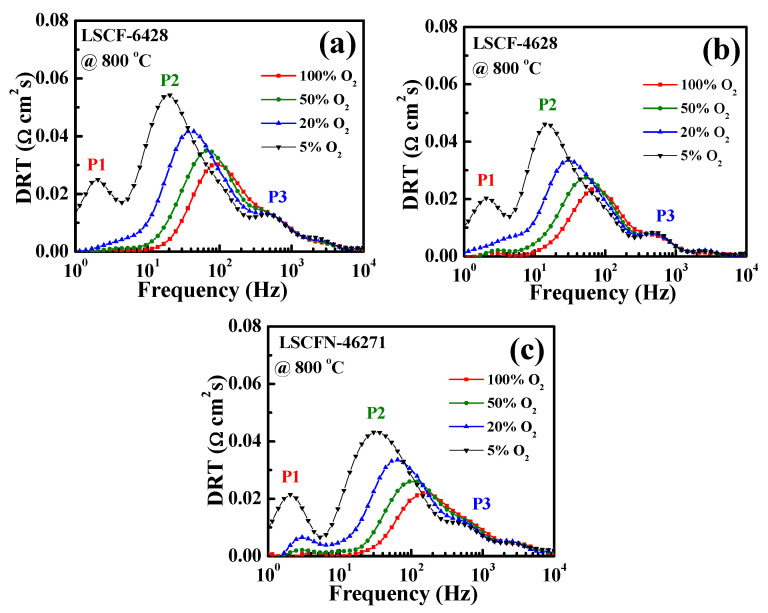
DRT plots for (**a**) LSCF−6428, (**b**) LSCF−4628, and (**c**) LSCFN−46271 electrodes measured at 800 °C under different oxygen partial pressures in O_2_ + N_2_ mixtures.

As illustrated in Figure 2, the distribution of relaxation time (DRT) was calculated based on the EIS data recorded at 800 °C. The electrode processes were arbitrarily divided into three distinct peaks, denoted as P1, P2, and P3, signifying the occurrence of three limiting sub-processes. P1 manifested when the O_2_ concentration dropped below 5%, with its appearance closely tied to oxygen concentration. Typically, the frequency range of 0–10 Hz is attributed to gas diffusion within and around the cathodes. The area beneath the P2 peaks displayed variability with oxygen partial pressure, and previous studies indicate its association with chemical reactions [29,30,31]. P2 was predominantly identified as a process linked to the chemical surface exchange of O_2_, featuring a frequency range of approximately 10–400 Hz. At higher frequencies, the DRT curves exhibited overlap, indicating that this region remained unaffected by fluctuations in O_2_ concentration. Consequently, P3 pertains to the electrode–electrolyte interface process and corresponds to charge transfer reactions at the triple phase boundary, encompassing a frequency range of 400–10,000 Hz. Notably, in Figure 2, P2 is characterized by the tallest peaks, suggesting that the surface exchange kinetics of oxygen at the cathode are likely the rate-determining reaction.

### 2.2. The Influence of CO_2_

The LSCF−6428, LSCF−4628, and LSCFN−46271 electrodes underwent testing at temperatures of 650 °C and 800 °C under various gas atmospheres, each featuring different CO_2_ concentrations. Figure 2 illustrates that augmenting the CO_2_ content in the air led to an increase in the electrode polarization resistance (Rp).

From these findings, it becomes evident that the performance alteration of (La,Sr)(Co,Fe)O_3-δ_ cathodes appears to be more responsive to CO_2_ at the lower temperature of 650 °C than at 800 °C. Specifically, for LSCF−6428 at 650 °C, Rp values of 0.510 Ω cm^2^ (air), 0.534 Ω cm^2^ (20% CO_2_-air), and 0.556 Ω cm^2^ (50% CO_2_-air) were recorded. In comparison, LSCF−4628 and LSCFN−46271 exhibited Rp values of 0.390 Ω cm^2^, 0.414 Ω cm^2^, and 0.443 Ω cm^2^ and 0.400 Ω cm^2^, 0.415 Ω cm^2^, and 0.430 Ω cm^2^, respectively, under the same gas atmospheres. Notably, the Rp increased with rising CO_2_ content in the air. Under identical atmospheric conditions, LSCF−6428 exhibited the highest Rp, while the difference between LSCF−4628 and LSCFN−46271 was relatively minor.

However, at 800 °C, Rp did not exhibit a clear trend under the three atmospheres. For instance, the initial impedances of the LSCF−6428, LSCF−4628, and LSCFN−46271 electrodes were approximately 0.050 Ω cm^2^, 0.039 Ω cm^2^, and 0.043 Ω cm^2^, respectively. Comparative analysis revealed that the Rp values of LSCF−4628 and LSCFN−46271 were lower than those of LSCF−6428.

**Figure 3:**
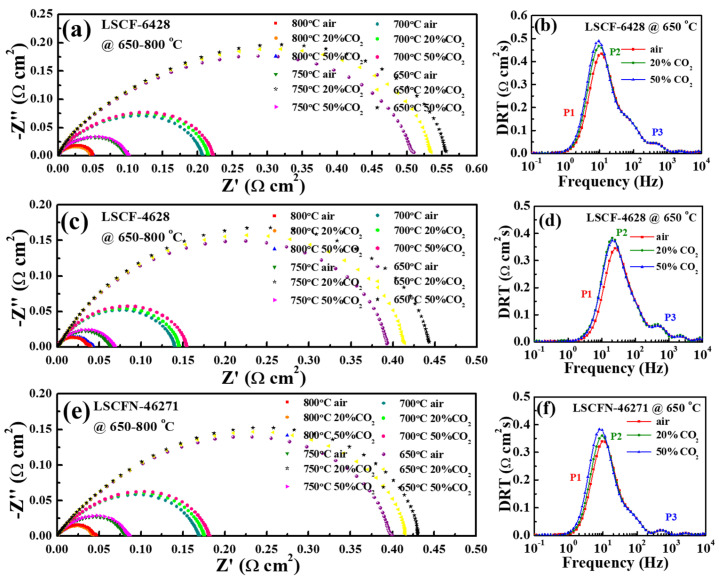
Polarization impedance spectra of LSCF−6428 (**a**), LSCF−4628 (**c**), and LSCFN−46271 (**e**) at 600–800 °C and different CO_2_ concentrations, and DRT plots of LSCF−6428 (**b**), LSCF−4628 (**d**), and LSCFN−46271 (**f**) at 650 °C.

Given the significant influence of CO_2_ content on EIS at 650 °C, DRT analysis was applied to these results. Figure 3b,d,f display the primary response frequencies. With increasing CO_2_ content, the P2 peaks shifted towards lower frequencies, and their area also expanded. As depicted in Figure 1, the P2 peak correlates with the surface exchange of O_2_. Consequently, it can be inferred that CO_2_ predominantly impacts the oxygen surface exchange reaction [32]. The observed degradation is likely attributed to the competition between CO_2_ and O_2_ for available oxygen vacancies, leading to a competitive mechanism that affects cathode performance.

### 2.3. Electrode Durability

**Figure 4:**
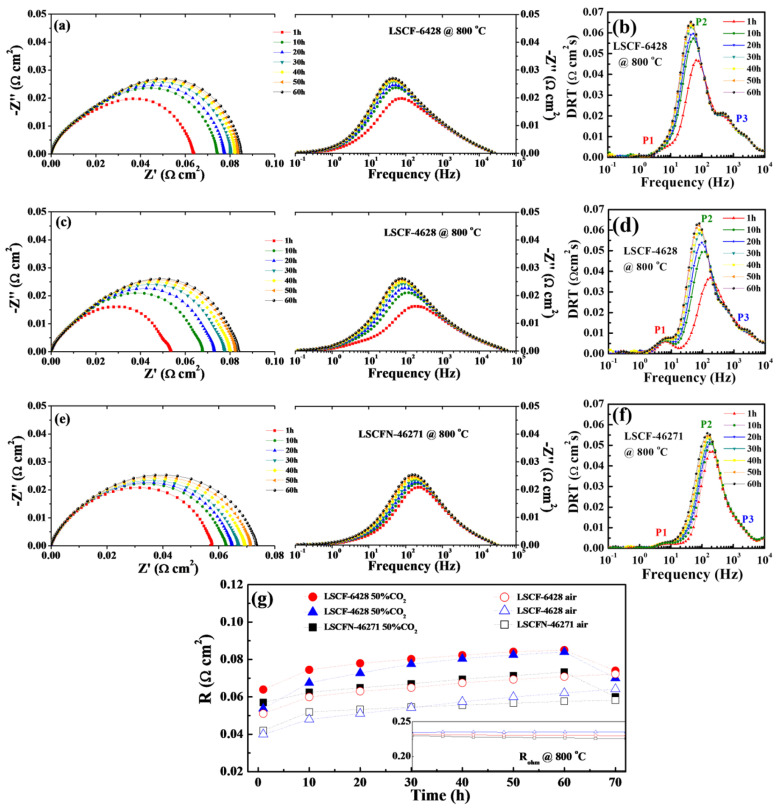
LSCF−6428 (**a**), LSCF−4628 (**c**), and LSCFN−46271 (**e**) impedance Nyquist and Bode tests in a 50% CO_2_-50% air atmosphere at 800 °C, and DRT plots for LSCF−6428 (**b**), LSCF−4628 (**d**), and LSCFN−46271 (**f**), (**g**) shows the polarization impedance numerical changes corresponding to a 70 h stability test for the 3 electrodes (inset displays ohmic impedance values).

The endurance of (La,Sr)(Co,Fe)O_3-δ_ cathodes when exposed to CO_2_ was further scrutinized at 800 °C under a 50% CO_2_-air atmosphere for durations of up to 60 h. Figure 4a,c,e depict the EIS and Bode spectra for LSCF−6428, LSCF−4628, and LSCFN−46271, respectively. Over time, the Rp values for all three electrodes exhibited an upward trend. Specifically, LSCF−6428 increased from ~0.064 Ω cm^2^ at 1 h to 0.085 Ω cm^2^ at 60 h. Similarly, LSCF−4628 and LSCFN−46271 increased from ~0.053 Ω cm^2^ to 0.084 Ω cm^2^ and ~0.057 Ω cm^2^ to 0.073 Ω cm^2^, respectively. The evolution of Rp values over time is illustrated in Figure 4g, indicated by solid symbols. Across the three (La,Sr)(Co,Fe)O_3-δ_ cathodes, the trends were remarkably similar, with all three Rp values showing a relatively rapid increase during the initial 10 h, followed by a more gradual rise. Notably, LSCF−4628 exhibited the most pronounced degradation rate.

The DRT analysis of the EIS results is illustrated in Figure 4b,d,f. The electrochemical processes associated with P1 and P3 remained relatively stable throughout the test duration. However, P2, corresponding to the oxygen adsorption and release process, shifted to lower frequencies over time, accompanied by an increase in peak area. Consequently, a plausible inference is that adsorbed CO_2_ partially covered the catalytically active sites of (La,Sr)(Co,Fe)O_3-δ_ for the oxygen reduction reaction, thus hindering the diffusion of O_2_.

To assess the magnitude of the CO_2_ effect, a baseline test was conducted. The three (La,Sr)(Co,Fe)O_3-δ_ cathodes were subjected to testing at 800 °C under 100% air for 70 h (i.e., not exposed to CO_2_). The results are presented in Figure 4g, indicated by hollow symbols. In the absence of CO_2_, the degradation rates of Rp were considerably lower than those observed under the 50% CO_2_-air mixed gas atmosphere. According to previous studies, the performance degradation of (La,Sr)(Co,Fe)O_3-δ_ cathodes over time might be attributed to Sr migration and segregation on the electrode surfaces at high temperatures [33].

For cathodes subjected to a CO_2_-rich environment, at the 60 h mark, the gas flow was reverted to atmospheric air for a duration of 10 h to assess the possibility of reversing the increase in Rp upon CO_2_ removal. EIS measurements demonstrated that the Rp values decreased and closely approximated those of the electrodes that had not been exposed to CO_2_. In other words, the electrode performance experienced a substantial recovery. This observation suggests that exposure to CO_2_ likely did not result in any permanent damage to the (La,Sr)(Co,Fe)O_3-δ_ cathodes. 

In the figure within Figure 4g, the impedance change over time of the three symmetrical electrodes is represented by the impedance evolution. It can be observed from the figure that the ohmic impedance remained almost constant during the 70 h stability test, and its influence can be neglected.

**Figure 5:**
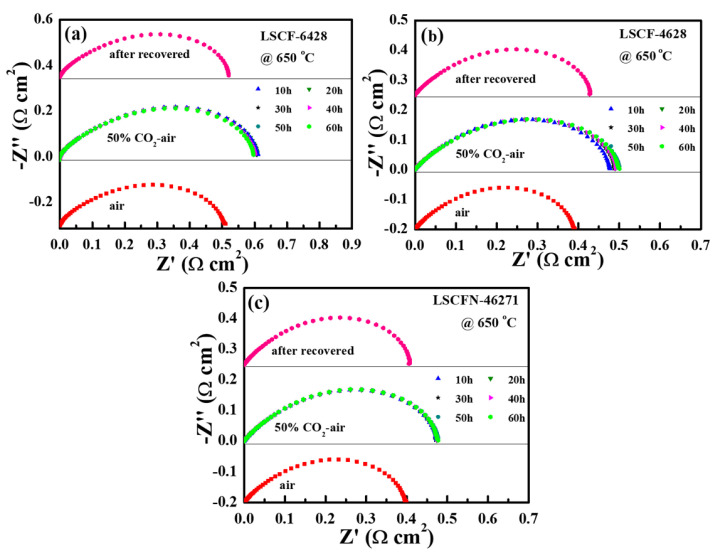
EIS stability test of LSCF−6428 (**a**), LSCF−4628 (**b**), and LSCFN−46271 (**c**) in a 50% CO_2_-air environment at 650 °C.

The corrosion stability of the (La,Sr)(Co,Fe)O_3-δ_ cathodes at 650 °C was also investigated, and the results are presented in Figure 5. The test commenced with the use of ambient air, after which the gas flow was transitioned from 100% air to a 50% CO_2_-air mixture, maintained for a duration of 60 h, before reverting to 100% air conditions. In the case of LSCF−6428, the Rp increased from 0.510 Ω cm^2^ to 0.611 Ω cm^2^ within the initial 10 h. Nevertheless, in comparison with EIS conducted at 800 °C, the impedance value did not exhibit significant alterations after 50 h. This observation suggests that LSCF−6428 demonstrated a higher degree of stability at lower temperatures. Upon replenishing the tube furnace with 100% air for 10 h, the EIS readings dropped back to approximately 0.520 Ω cm^2^. Similar trends were observed for cathodes LSCF−4628 and LSCFN−46271. To better illustrate the changes in impedance values, the impedance variations during the process are presented in Table 1. It becomes evident that the degradation patterns of the LSCF cathodes at 650 °C resembled those at 800 °C. Importantly, the exposure to CO_2_ did not lead to permanent damage to the electrodes, as the electrode performance could be readily restored upon CO_2_ removal.

This CO_2_-induced performance degradation is most likely attributable to the competition between CO_2_ and O_2_ molecules vying for active sites on the electrode surface during oxygen exchange reactions [34,35].

### 2.4. XRD Analysis

**Figure 6:**
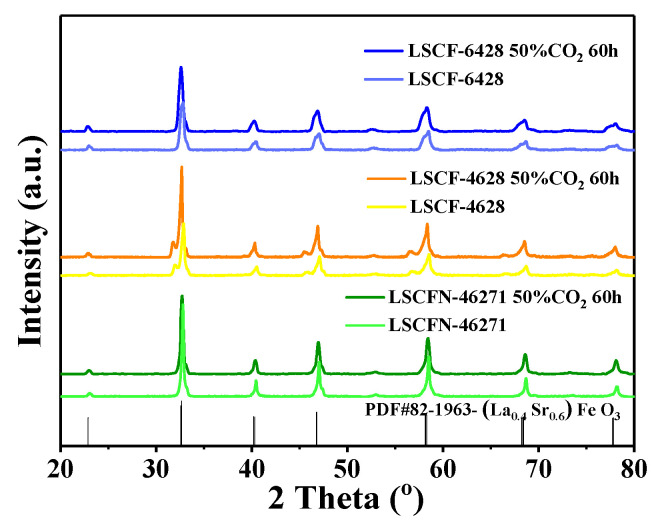
XRD patterns of the three cathodes before and after testing at 800 °C in 50% CO_2_-air for 60 h.

XRD patterns of the (La,Sr)(Co,Fe)O_3-δ_ cathodes, both before and after annealing in a 50% CO_2_-air environment at 800 °C, are illustrated in Figure 6. Through a comparative analysis with the reference spectra obtained without heat treatment and the standard PDF cards (PDF#82-1963), it can be unequivocally concluded that all three (La,Sr)(Co,Fe)O_3-δ_ cathodes retained their singular-phase perovskite structure with a cubic lattice. No evidence of any additional impurity phases was detected, or if present, their content was below the detection limit of the XRD analysis. These findings serve as strong indicators that all three cathodes remained stable when exposed to a CO_2_-containing atmosphere and did not undergo any reactive interactions with CO_2_ during the testing period.

### 2.5. SEM Analysis

**Figure 7:**
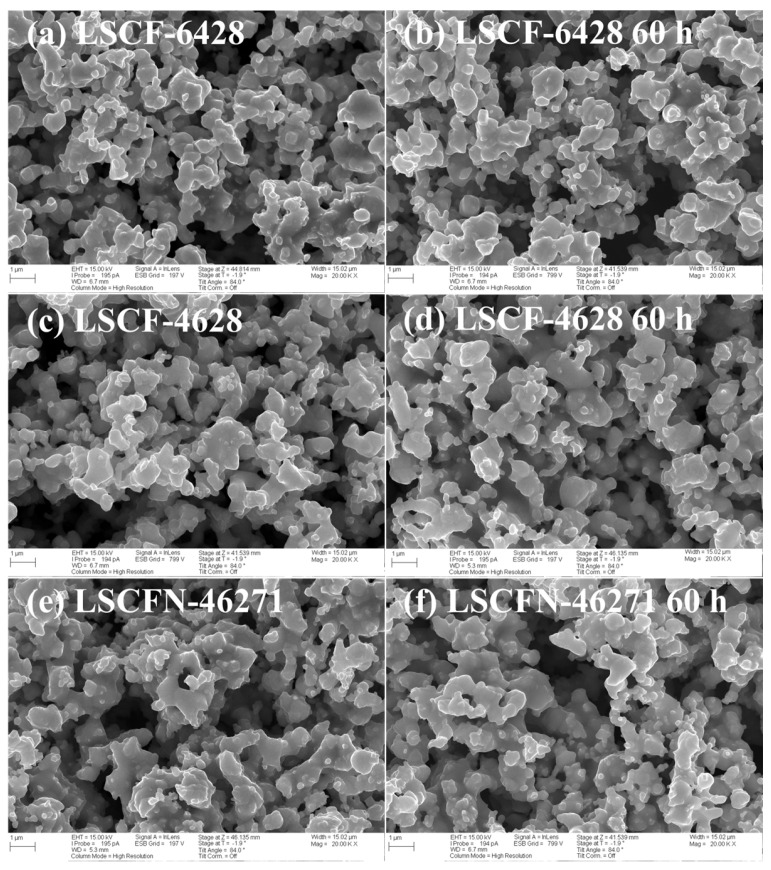
SEM micrographs on the surfaces of (**a**,**b**) LSCF−6428, (**c**,**d**) LSCF−4628, and (**e**,**f**) LSCFN−46271 before and after 60 h corrosion in 50% CO_2_-air at 800 °C.

The morphological evaluation of the (La,Sr)(Co,Fe)O_3-δ_ cathodes, both prior to and after the CO_2_ stability testing, is depicted in Figure 7. SEM images of the LSCF electrode surfaces before the testing phase are displayed in Figure 7a,c,e, corresponding to LSCF−6428, LSCF−4628, and LSCFN−46271, respectively. These electrodes were characterized by fine particle structures with a high degree of porosity, ensuring efficient gas diffusion. Notably, the surface morphologies of all three cathodes exhibited striking similarities.

Figure 7b,d,f present SEM images of the LSCF electrode surfaces after being subjected to 60 h exposure to 50% CO_2_-air at 800 °C. A comparison of these two sets of images clearly indicates that there was virtually no discernible alteration in electrode porosity or particle size following the 60 h CO_2_ exposure period. The combined results from XRD and SEM provide compelling evidence that the LSCF−6428, LSCF−4628, and LSCFN−46271 cathodes remained stable when exposed to high-temperature, high-CO_2_ atmospheres. Importantly, they did not undergo any reactive interactions with CO_2_ throughout the testing process, and their surfaces remained smooth and devoid of impurities at the conclusion of the test.

## 3. Experimental Section

### 3.1. Material Synthesis and Electrode Fabrication

La_0.6_Sr_0.4_Co_0.2_Fe_0.8_O_3-δ_ (LSCF−6428), La_0.4_Sr_0.6_Co_0.2_Fe_0.8_O_3-δ_ (LSCF−4628), and La_0.4_Sr_0.6_Co_0.2_Fe_0.7_Nb_0.1_O_3-δ_ (LSCFN−46271) cathode powders were synthesized using the solid-phase synthesis method. Details regarding the material selection, preparation procedures, and the formulation of the electrode ink can be found in our prior publication [20]. As the electrolyte, a gadolinia-doped ceria (GDC) substrate with a thickness of 150 μm was employed, sourced from Kerafol Keramische Folien GmbH, Germany. Subsequently, the GDC substrate was precisely laser-cut into square pieces measuring 9 mm×9 mm.

For the measurement of electrochemical impedance spectroscopy (EIS), symmetric (La,Sr)(Co,Fe)O_3-δ_ electrodes were meticulously fabricated. (La,Sr)(Co,Fe)O_3-δ_ powder was admixed with a slurry composed of pine oil alcohol and ethyl cellulose. LSCF−6428, LSCF−4628, and LSCFN−46271 electrode inks were screen-printed on both sides of the GDC substrates, followed by sintering in air at 1100 °C for 2 h. The thickness of (La,Sr)(Co,Fe)O_3-δ_ and GDC was about 30 μm and 150 μm. This process yielded three distinct types of symmetric electrodes: LSCF−6428||GDC||LSCF−6428, LSCF−4628||GDC||LSCF−4628, and LSCFN−46271||GDC||LSCFN−46271. The active area of the electrodes was 0.81 cm^2^.

### 3.2. Material Characterization

Initially, the symmetric electrodes underwent testing under varying oxygen partial pressures within N_2_ + O_2_ mixtures at 800 °C, followed by exposure to air containing different levels of CO_2_ at temperatures ranging from 650 °C to 800 °C. To assess changes resulting from these tests, the phase compositions of the electrodes were examined using a Bruker D8 Advance X-ray diffractometer (XRD). Additionally, the microstructure of the sample surfaces was characterized through the utilization of a ZEISS Merlin scanning electron microscope (SEM).

The electrochemical impedance spectroscopy (EIS) responses of the symmetric electrodes subjected to various atmospheres were investigated using a Solartron 1260 instrument employing a signal amplitude of 10 mV across a frequency range of 10^−1^–10^6^ Hz, and the bias potential was set to 0. The symmetric electrodes were placed inside a controlled-atmosphere furnace, with a collector layer consisting of gold paste and silver wires serving as conductive leads. During the measurement, various gases were introduced to the symmetric electrodes at a flow rate of 6 L/h.

**Figure 8:**
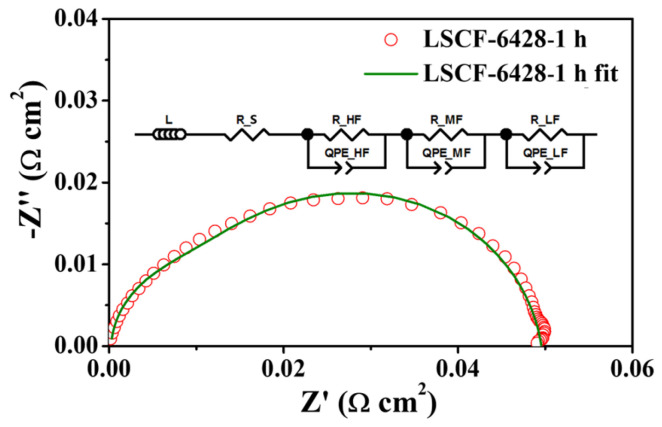
EIS at 800 °C in air for LSCF−6428, fitting results, and fitted equivalent circuit diagram.

Figure 8 illustrates the EIS data for LSCF−6428 at 1 h, as shown in Figure 4a, along with the corresponding EIS fitting diagram. The equivalent circuit model for the symmetric electrode consists of an inductor (L), a resistance element (Rs), and three R-CPE components. L and Rs primarily represent the inductance of the conductor and the overall ohmic impedance. R_HF_, R_MF,_ and R_LF_ correspond to the high-frequency, mid-frequency, and low-frequency polarization resistances, respectively. The total cell Rp is the sum of these three components. The fitting results of this equivalent circuit in the figure closely match the experimental data [36]. Using impedance spectra to calculate the area-specific resistance (ASR) is a method used to evaluate the electrochemical performance of symmetric electrodes. The ohmic resistance is adjusted to zero, and due to the symmetrical unit structure, the calculated values are divided equally to obtain the ASR value for a single electrode [37].

DRT analysis entails fitting complex impedance data to a relaxation time distribution. The fundamental concept behind this technique is to disentangle the impedance spectrum into a sequence of relaxation processes. The Kramers–Kronig transformation establishes a mathematical integral relationship between the real and imaginary components of impedance. The general mathematical expression for this transformation is expressed as follows:

DRT(τ) = −2/π ∫ [Z′(ω) − Z′∞]/ω f(τ/ω) dω,

DRT(τ) signifies the distribution of relaxation times as a function of τ, which represents the relaxation time. Z′(ω) denotes the real component of impedance with respect to angular frequency (ω). Z′∞ corresponds to the high-frequency limit of the real impedance. f(τ/ω) is a weighting function employed in the analysis. The identification of characteristic peaks within the DRT spectrum was conducted through a comprehensive analysis. Variations in the DRT peaks, as an integral part of the relaxation time spectrum, were associated with distinct electrochemical processes. Impedance data analysis was carried out using the rapid and versatile data analysis/visualization (Ravdav) software version 0.9.7 [38].

## 4. Conclusions

In this research, we conducted a comprehensive investigation into the performance and stability of three distinct (La,Sr)(Co,Fe)O_3-δ_ cathodes when exposed to CO_2_-enriched air environments. Utilizing DRT analysis, we successfully identified three distinct electrochemical processes along with their corresponding frequency ranges. Under CO_2_-containing atmospheres at temperatures ranging from 650 °C to 800 °C, EIS measurements revealed a consistent increase in impedance across all three cathodes as CO_2_ concentrations increased. This phenomenon was attributed to the adsorption of CO_2_ molecules onto the active oxygen sites present on the electrode surfaces, leading to a reduced oxygen exchange rate and, consequently, an increase in Rp.

Crucially, XRD and SEM analyses confirmed the absence of any adverse reactions or corrosion of the electrodes induced by CO_2_ exposure. No secondary phases were detected, demonstrating the resilience of the cathode materials to CO_2_-induced corrosion or degradation. This suggests that (La,Sr)(Co,Fe)O_3-δ_ cathodes do not undergo chemical reactions with CO_2_ and maintain their structural integrity during exposure to CO_2_-enriched atmospheres.

Furthermore, when the gas supply was reverted to 100% air, the recorded EIS spectra indicated a remarkable recovery in electrode performance. This recovery can be attributed to the removal of CO_2_ from the electrode surface, allowing the active oxygen sites to become available for oxygen exchange reactions once again. In essence, our findings reveal that the performance deterioration observed in (La,Sr)(Co,Fe)O_3-δ_ electrodes when exposed to CO_2_ is entirely reversible upon CO_2_ removal, highlighting the excellent corrosion resistance exhibited by these cathodes in the face of CO_2_ exposure.

Among the three different cathode compositions examined in this study, it is noteworthy that LSCF−6428 and LSCFN−46271 displayed superior durability compared to LSCF−4628. Both LSCF−6428 and LSCFN−46271 emerge as promising cathode materials for SOFCs.

## Figures and Tables

**Table 1:** Polarization impedance values during the transition between air and 50% CO_2_-air.

Electrode	Air/Ω cm^2^	50% CO_2_-Air 10 h/Ω cm^2^	50% CO_2_-Air 30 h/Ω cm^2^	50% CO_2_-Air 60 h/Ω cm^2^	After Recovered/ Ω cm^2^
LSCF−6428	0.510	0.611	0.601	0.597	0.520
LSCF−4628	0.392	0.478	0.488	0.501	0.427
LSCFN−46271	0.400	0.475	0.472	0.478	0.406

## Data Availability

This study did not involve the creation of new data, and it did not include original data that can be shared. Data sharing is constrained by privacy and ethical considerations. Therefore, we are unable to provide additional data support. We acknowledge the importance of data sharing in scientific research and encourage future studies to actively seek data sharing when possible. If there are any data requests or inquiries, please feel free to contact us, and we will make every effort to provide further information about the research.

## Supplementary Materials

The following supporting information can be downloaded at: https://www.mdpi.com/article/10.3390/molecules28227490/s1.
