# Supplementary material for: Mechanistic Study on the Corrosion of (La,Sr)(Co,Fe)O3-δ Cathodes Induced by CO2
Source: Molecules. 2023 Nov 8;28(22):7490. doi: 10.3390/molecules28227490 (PMC10673451; doi:10.3390/molecules28227490)
Supplement: Supplementary file 1 [file molecules-28-07490-s001.zip › molecules-2581538-supplementary material.pdf]

Supporting Information

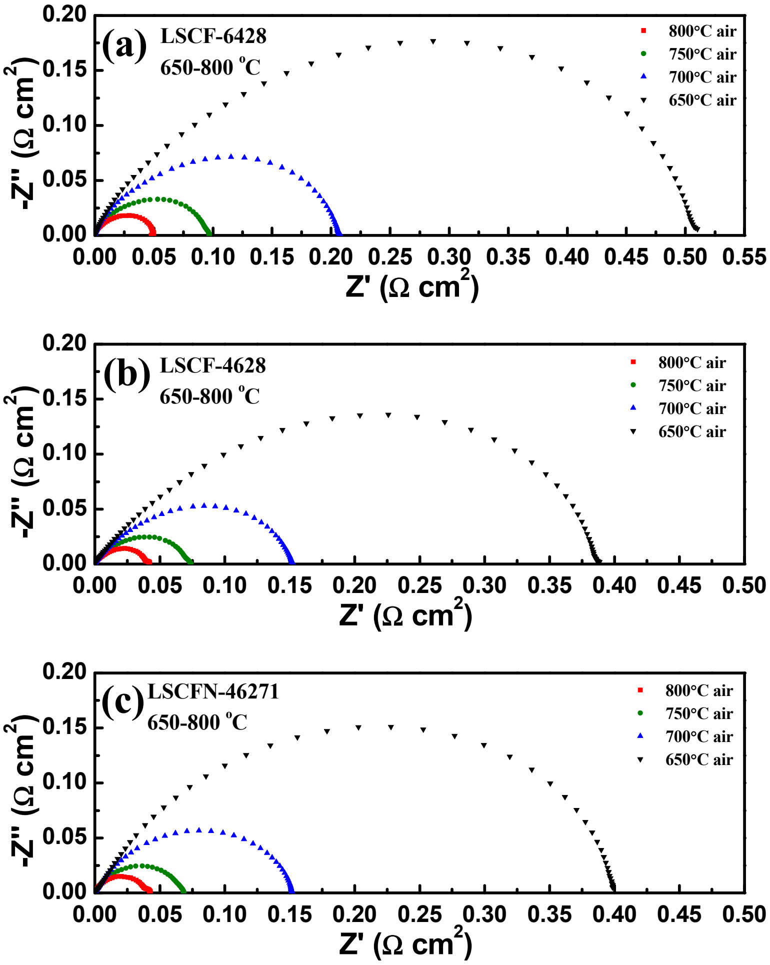

Figure S1. EIS of 3 symmetric electrodes between 650-800 °C in air.

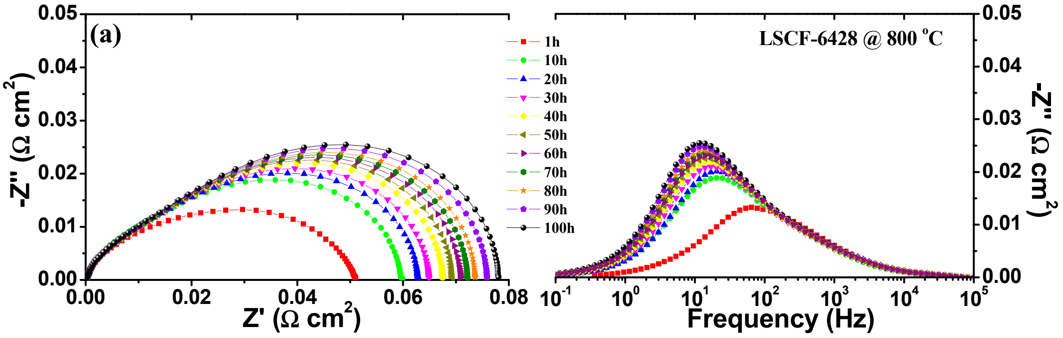

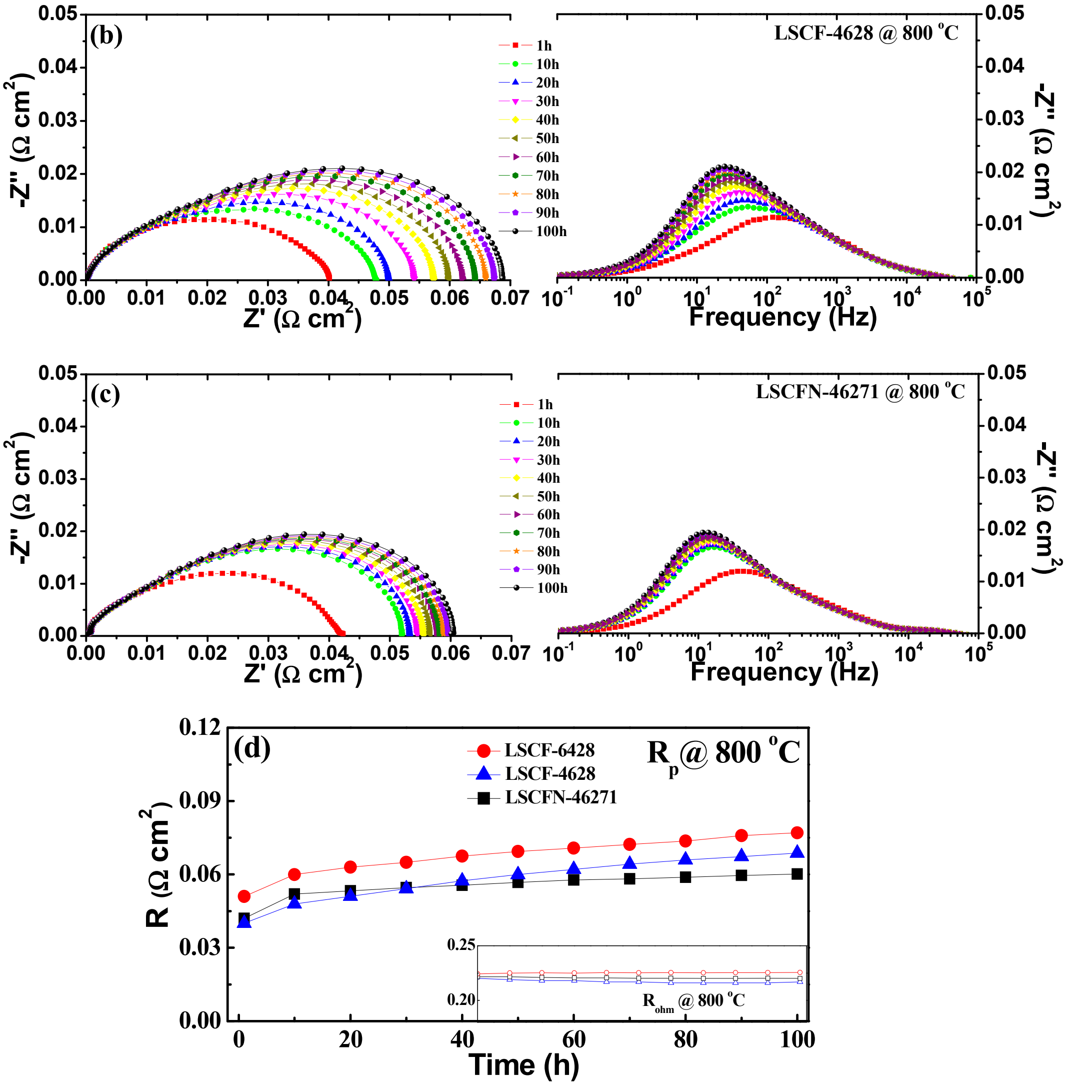

Figure S2. EIS stability test of 3 symmetrical electrodes in air at 800 °C.

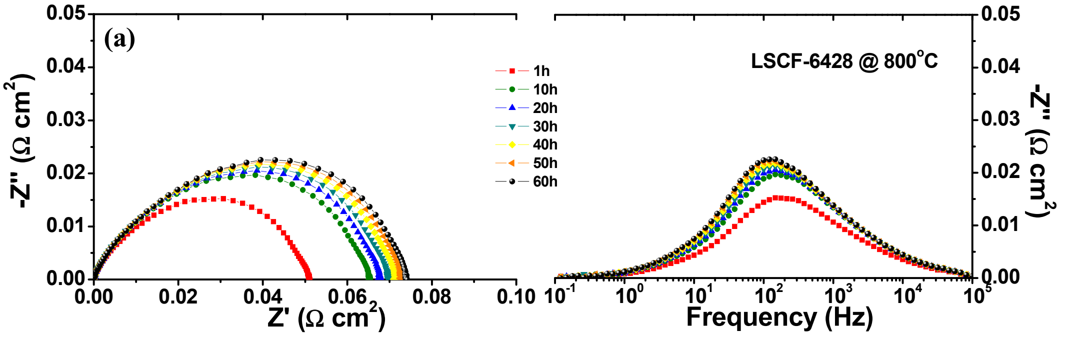

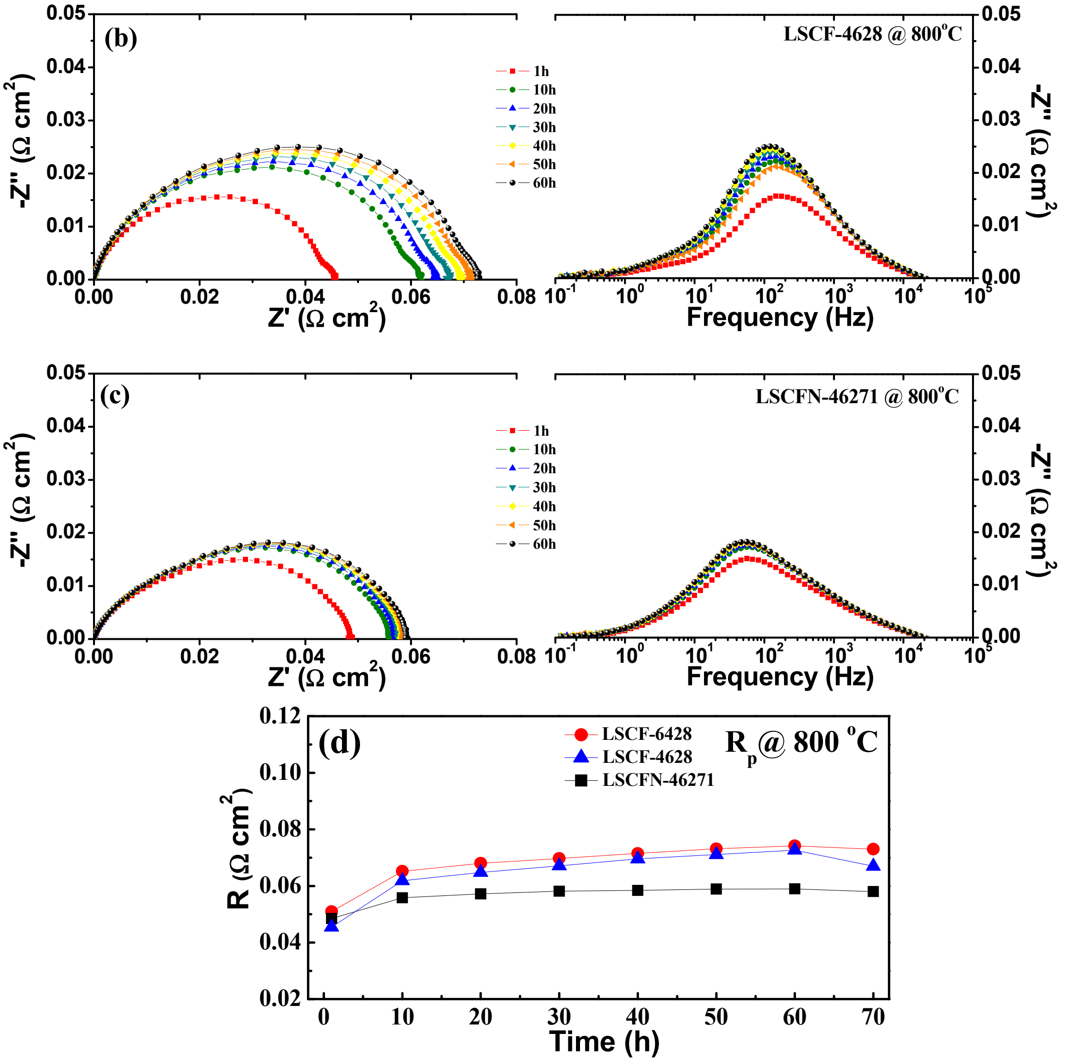

Figure S3. EIS test of the stability of 3 symmetrical electrodes in 20% CO_2-80% Air at 800 ºC.

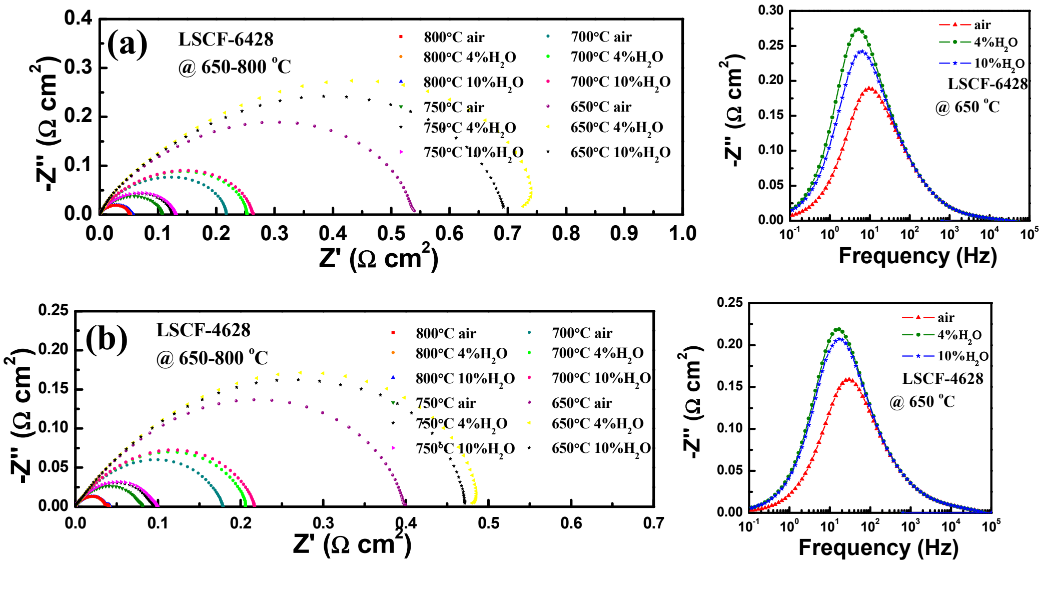

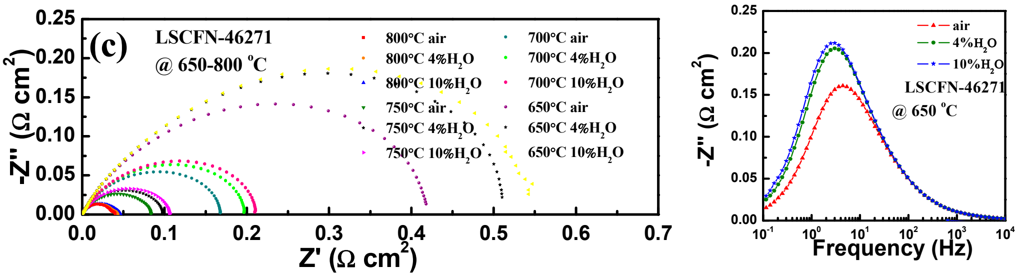

Figure S4. Nyquist plots and Bode plots of 3 electrodes under different H_2O concentration at 650-800 °C.

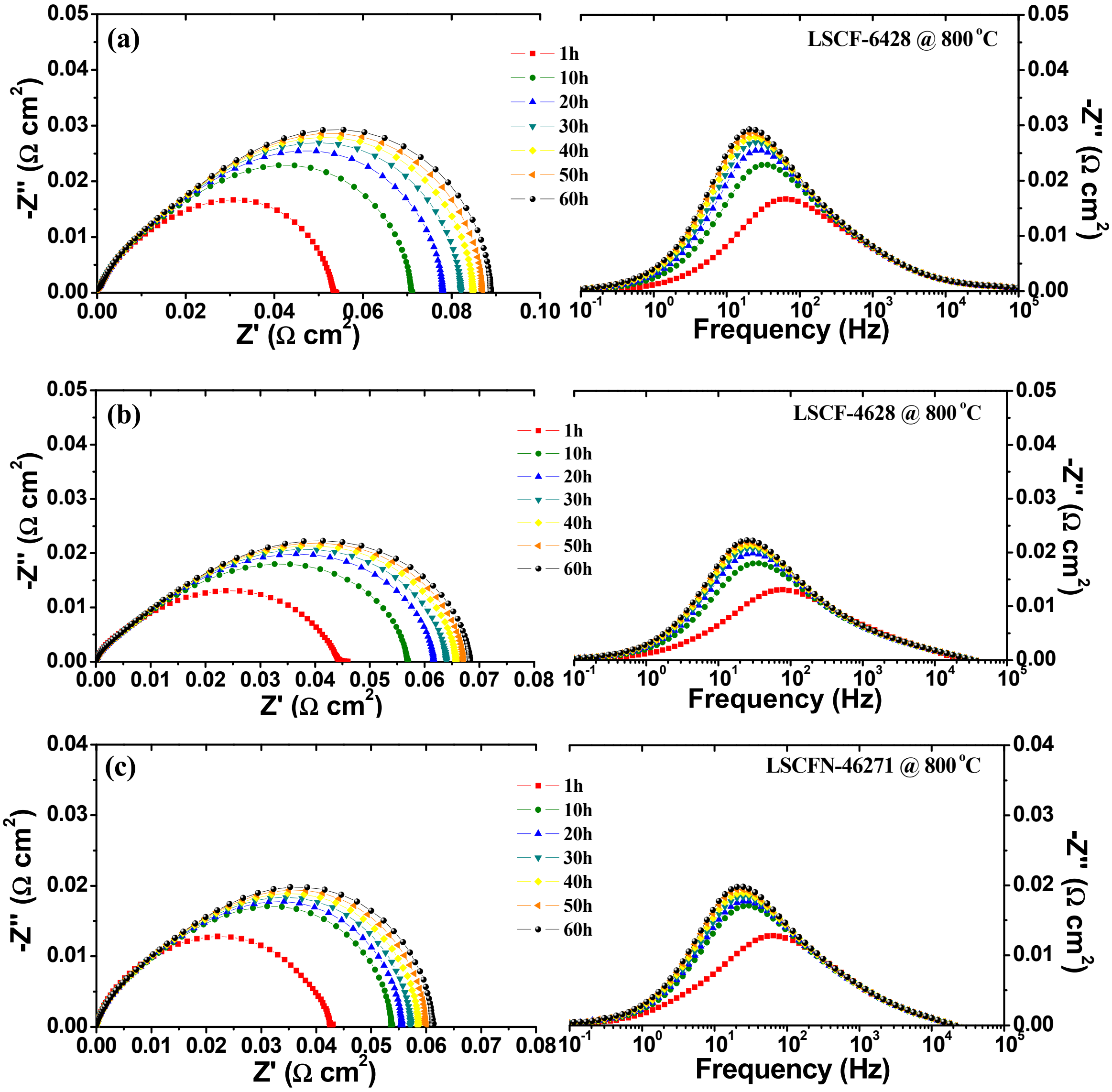

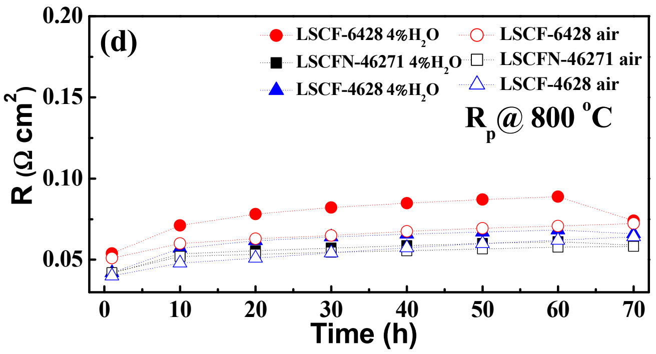

Figure S5. EIS stability test of 3 symmetrical electrodes in 4% H_2O-96% Air at 800 ºC.

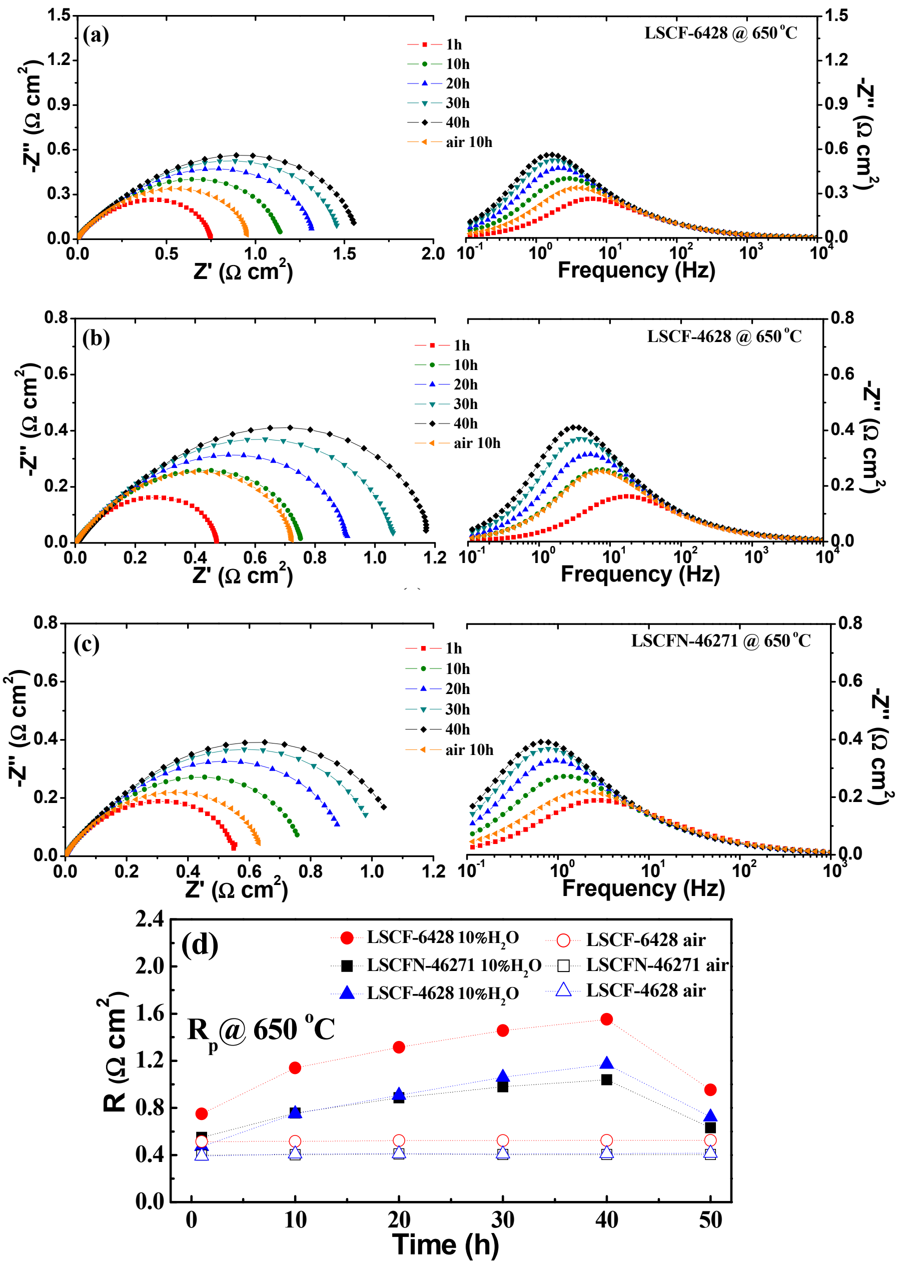

Figure S6. EIS stability test of 3 symmetrical electrodes in 10% H_2O-90% Air at 650 ºC.
